# Toll-like receptors (TLRs) expression and function in response to inactivate hyphae of *Fusarium solani* in immortalized human corneal epithelial cells

**Published:** 2007-10-17

**Authors:** Xiuming Jin, Qin Qin, Lili Tu, Xiangtian Zhou, Yi Lin, Jia Qu

**Affiliations:** 1 Eye Center, Affiliated Second Hospital, School of Medicine, Zhejiang University, Hangzhou, China;; 2 School of Ophthalmology and Optometry, Eye Hospital, Wenzhou Medical College, Wenzhou, Zhejiang, China

## Abstract

**Purpose:**

To evaluate the role of toll-like receptors (TLRs) in host responses to *Fusarium solani* by the use of cultured immortalized human corneal epithelial cells (HCEC) and to determine whether inactive hyphal fragments can induce an antifungal response in these cells.

**Methods:**

Cultured HCEC cells were stimulated with inactive hyphal fragments from *Fusarium solani*, and the effect on expression of TLRs was determined by real-time polymerase chain reaction (PCR), immunofluorescence, and western blot analysis. Cells were also cocultured with hyphal fragment and hydrocortisone to determine whether hydrocortisone modulates the transcription of TLRs. The release of interleukin-6 (IL-6) and IL-8 was also measured using enzyme-linked immunosorbent assays (ELISA) in the presence and absence of specific blocking antibodies to TLR2 and TLR4.

**Results:**

Incubation of HCECs with inactive hyphal fragments upregulated the expression of TLR2, 3, 4, and 6 mRNAs and increased the release of IL-6 and IL-8. Immunofluorescence staining and western blot analysis confirmed that expression of TLR2 and TLR4 was upregulated in response to hyphal fragments. This upregulation was further enhanced by cotreatment with hydrocortisone. Results from ELISA assays showed that the concentration of IL-6 was increased, and the concentration of IL-8 was decreased in supernatants of HCECs after treatment with both hydrocortisone and hyphal fragments. The release of IL-6 and IL-8 was also inhibited by incubation with anti-TLR2 and anti-TLR4 monoclonal antibodies.

**Conclusions:**

HCECs are involved in the cornea immune response to fungal infections. TLR2 and TLR4 may play a crucial signaling role in response to *Fusarium* hyphae in HCECs. Glucocorticoids such as hydrocortisone can enhance the expression of the TLRs on the epithelium and thus may enhance the resistance to fungal infections in the cornea. These findings may provide crucial information for understanding the immune mechanisms of fungal keratitis and promote the design of new immune therapeutical approaches to fungal keratitis.

## Introduction

Mycotic keratitis is an opportunistic fungal infection of the cornea caused by either a reduction in the local defense mechanism or by injury to the corneal epithelium. If not correctly diagnosed and treated, mycotic keratitis can result in a marked loss of vision and eventually cause a complete perforation of the cornea. Members of the genus *Fusarium* are the most frequently isolated fungi in patients with fungal keratitis. Furthermore, *Fusarium* has increasingly been identified in deep tissues and disseminated infections [1-3]. Despite the known existence of many commensal fungi within the conjunctiva sac, the cornea is not normally inflamed. However, the mechanism by which the host can successfully defend against invasive fungi is still unknown.

The corneal epithelium is known to be an efficient physical barrier to infections and constitutes the first line of defense against microbial pathogens [4]. This is why fungal keratitis always occurs after the epithelial integrity of the cornea has been breached, exposing underlying fibroblasts [5-8]. The ability of epithelial cells to detect various pathogens and to tailor their response to a specific pathogen is critical for the induction of innate immunity and for the establishment of adaptive immune responses [9]. In addition, vasculature does not exist in the cornea under normal conditions, and the cells and molecules operating during early stages of the immune response have a decisive impact on the shaping of the subsequent adaptive response in the cornea. Understanding the role that epithelial cells play in immune surveillance and host defense is crucial for understanding the occurrence, progression, and diagnosis of fungal keratitis and for developing new and effective therapies to combat this infection.

Recent studies indicate that recognition of pathogens is largely assigned to an evolutionarily conserved family of receptors known as toll-like receptors (TLRs). These receptors function in innate immunity via the recognition of pathogen-associated molecular patterns [10,11]. Previous studies showed that human corneal epithelial cells express several different TLRs and are able to recognize various pathogens including Gram-positive bacteria [12], Gram-negative bacteria [13,14], and viruses [15,16]. These TLRs respond to pathogen-associated patterns such as flagellin, lipopeptides, and poly(I:C). Recent studies have also demonstrated that toll-like receptors (TLRs) are crucial for the recognition of fungal pathogens such as *Candida albicans* [17-19], *Aspergillus fumigates* [20-24], and *Cryptococcus neoformans* [25-27]. However, no reports have yet investigated the relationship between TLRs and *Fusarium*. To better understand the innate immune response of corneal epithelial cells to fungal infections, we investigated the role of TLRs in immortalized human corneal epithelial cells (HCEC) in response to inactive hyphal fragments from *Fusarium solani*.

## Methods

### Reagents and antibodies

Dulbecco's Modified Eagle Medium (DMEM), F12, fetal bovine serum (FBS), and phosphate-buffered saline (PBS) were obtained from Invitrogen-Gibco (New York, NY). All media and cytokines used for cell culture were endotoxin minimized. Tissue culture dishes and six-well chamber slides were from BD (New York, NY). Hydrocortisone was obtained from Calbiochem (Darmstant, Germany). Affinity purified, monoclonal, anti-human TLR2, TLR4, and normal mouse immunoglobulin G (IgG) were from eBioscience (San Diego, CA). CY3-conjugated secondary antibody was from Beyotime Biotechnology (Beyotime, China). 2-(4-Amidinophenyl)-6-indolecarbamidine dihydrochloride (DAPI dihydrochloride) was used to dye nuclei and was purchased from Beyotime. Paired antibodies for human interleukin-6 (IL-6) and IL-8 enzyme-linked immunosorbent assays (ELISA) were from BD. RNeasy Mini kits were purchased from Qiagen (Valencia, CA) for RNA extraction. RNA polymerase chain reaction (PCR) kits were from Promega (Fitchburg, WI), and ethidium bromide, DNA molecular size markers, and agarose were from Gene Tech (Shanghai, China). SYBR Green PCR kits were from Applied Biosystems (Foster City, CA).

### Isolation of killed hyphal fragments

**Figure 1:**
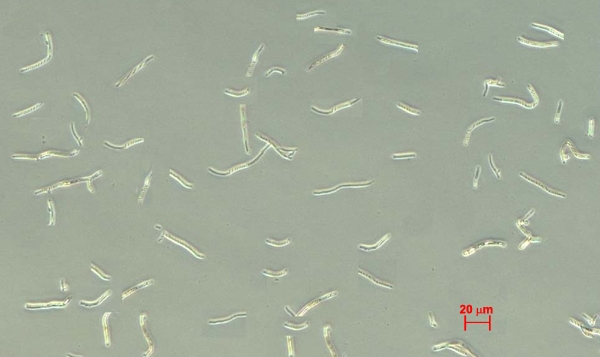
Morphology and size of the killed hyphal fragments

The *Fusarium solani* strain (serial number: 3.2889) was purchased from the China General Microbiological Culture Collection Center (CGMCC, Beijing, China). Isolation and preparation of killed hyphal fragments was previously described [20,21,28]. In brief, the strains were grown on Sabouraud glucose agar supplemented with penicillin-streptomycin for four to seven days at 35 °C. Conidia were harvested by gently scraping the surfaces of the slants and suspending them in PBS. To suspend the very hydrophobic conidia of *Fusarium solani*, 0.05% Tween 80 was added to the PBS. To remove hyphae and debris, the conidial suspension was filtered through eight layers of cheesecloth. Conidia were cultured in YAPD (2% peptone, 2% glucose, 1% yeast extract, 0.01% adenine). After 48 h, more than 95% of blastoconidia were grown to hyphae, which were checked by microscope. To get hyphal fragments of a uniform size, hyphal fragments were treated with Micro Tissue Grinders and an Ultrasonic Cell Cracker (JY92–2D; Scientz Biotechnology Co, Ningbo, China). Hyphal fragments were then filtered through 400 mesh grit (38 μm diameter). Hyphal fragments were heat-killed for 15 min at 100 °C.The sterilized hyphae were centrifuged and resuspended vigorously in PBS, containing 10 mg of RNase A per ml, and incubated for 30 min at 37 °C to remove intracellular RNA. Finally, the hyphal fragments were washed three times with Hank's Buffered Salt Solution (HBSS) and stored at −80 °C. Microscopically, the morphology of the killed hyphal fragments (Figure 1) appeared to be intact (20–40 μm) and were counted on a hemacytometer. The killed hyphal fragments were always confirmed by culture in Sabouraud glucose broth.

The killed hyphal fragments were used to stimulate cultured cells in the experiment. The hyphal fragments appear to have an intact morphology with diameters of approximately 20–40 μm.

### Culture of immortalized human corneal epithelial cells

Simian virus (SV) 40 immortalized human corneal epithelial cells (HCEC) were cultured in DMEM/F12, supplemented with 10% FBS, 5 μg/ml insulin, 0.1 mu g/ml cholera toxin, 5 ng/ml human epidermal growth factor, and 40 μg/ml gentamicin at 37 °C under 95% humidity and 5% CO_2_ as described by Araki-Sasaki et al. [29]. The cells were kindly provided by Dr. Zan Pan (New York University, New York, NY).

### Cell challenge

The HCECs were seeded onto Laboratory-Tek tissue culture chamber slides for 24 h and then subjected to one of the following treatments: (1) treated with hyphal fragments (cell/hyphal is equivalent to 1/1) for 48 h at 37 °C; (2) treated with hydrocortisone (10 μg/ml) for 48 h at 37 °C; or (3) incubated with hydrocortisone (10 μg/ml) for 2 h at 37 °C followed by treatment with hyphal fragments (cell/hyphal is equivalent to 1/1) for 48 h at 37 °C. Following treatment, supernatants were collected and stored at −80 °C to evaluate the release of IL-6 and IL-8. mRNA was also extracted from cells following treatment to evaluate the expression of TLRs.

TLR blocking experiments were conducted by incubating HCECs with monoclonal antibodies (mAbs) against TLRs. HCECs were incubated at room temperature with anti-TLR4, anti-TLR2, both anti-TLR4 and anti-TLR2, or IgG control antibody for 1 h. Cells were then treated with hyphal fragments for 48 h at 37 °C, and the supernatants were collected to evaluate the releases of IL-6 and IL-8.

### Reverse transcription polymerase chain reaction

Total RNA prepared from confluent monolayers of HCEC was used to evaluate the expression of TLR mRNA. Qiagen RNeasy Mini kits were used for RNA extraction. Total RNA samples were then reverse transcribed in a final volume of 20 μl containing 1 μg RNA, 50 mM Tris-HCl, 75 mM KCl, 3 mM MgCl_2_, 5 mM dithiothreitol, 20 U of RNase Inhibitor, 1 μl (1 μg) random primer, 0.5 mM each dNTP, and 200 U RNase H-free reverse transcriptase (RT; Promega). The following primer pair was used for the analysis of GAPDH by reverse-transcription polymerase chain reaction (RT–PCR): 5′-CGG AGT CAA CGG ATT TGG TCG TAT-3′ and 5′-AGC CTT CTC CAT GGT TGG TGA AGA C-3′.The annealing temperature of the primers was 60 °C. Each PCR was performed in a 20 μl solution containing 1 μl RT reaction products, 10X PCR buffer 2 μl, 1.8 mM MgCl_2_, 0.1 mM each dNTP, 0.1 μM of upstream primer, 0.1 μM of downstream primer, and 0.5 U Taq DNA polymerase. A negative control (the PCR without a preceding RT step) for each sample was run to assess whether there was residual genomic DNA in the DNase-treated samples.

### Real-time polymerase chain reaction

Real-time PCR was performed in an ABI PRISM 7500 Sequence Detection System Thermal Cycler (Applied Biosystems). Real-time PCR was performed on a volume of 15 μl containing 1.5 μl (50 ng) of cDNA and 13.5 μl of master mix containing 7.5 μl of mix (SYBR Green PCR Master Mix, Applied Biosystems), 0.75 μl of each primer (10 pmol/l), and 4.5 μl of diethyl pyrocarbonate-treated water. The primers are listed in Table 1. The program was set at 50 °C for 2 min and 95 °C for 10 min followed by 40 cycles of denaturation at 95 °C for 15 s and annealing at 60 °C for 60 s. The melting curve was analyzed by elevating the temperature from 60 °C to 95 °C while monitoring fluorescence. SYBR green fluorescence was monitored after each elongation period. The housekeeping gene, GAPDH, was found to be constantly expressed in cornea. Samples were amplified with GAPDH primers for determination of the initial relative quantity of cDNA in each sample, and all PCR products were normalized to that amount. Negative controls (without template) were produced for each run.

**Table 1:** Primers for real-time polymerase chain reaction

Target gene	Locus	Forward sequence(5′-3′)	Reverse sequence (5′-3′)	Amplicon size (bp)
TLR1	NM003263	GCCCAAGGAAAAGAGCAAAC	AAGCAGCAATATCAACAGGAG	135
TLR2	NM003264	TCTCCCATTTCCGTCTTTTT	GGTCTTGGTGTTCATTATCTTC	125
TLR3	NM003265	TAAACTGAACCATGCACTCT	TATGACGAAAGGCACCTATC	101
TLR4	NM003266	GAAGCTGGTGGCTGTGGA	GATGTAGAACCCGCAAG	213
TLR5	NM003268	TTGCTCAAACACCTGGACAC	CTGCTCACAAGACAAACGAT	148
TLR6	NM006068	GTGCCATTACGAACTCTA	TTGTTGGGAATGCTGTT	109
TLR7	NM016562	CTGACCACTGTCCCTGAG	AACCCACCAGACAAACCA	263
TLR8	NM016610	AACATCAGCAAGACCCAT	GACTCCTTCATTCTCCCT	64
TLR9	NM017442	CGCCAACGCCCTCAAGACA	GGCGCTTACATCTAGTATTTGC	79
TLR10	NM030956	CTCCCAACTTTGTCCAGAAT	GGTGGGAATGCAATAGAAT	132
GAPDH		CCCCACACACATGCACTTACC	TTGCCAAGTTGCCTGTCCTT	100

Specific primers for TLR1–10 mRNAs were designed using Prime 3 software, possibly to be 1-intron spanning.

Samples were amplified in triplicate, averages were calculated, and differences in C_T_ data were evaluated by Sequence Detection Software V1.3.1 (Applied Biosystems). For data analysis, we used the comparative C_T_ method (ΔΔC_T_ method) with the following formula: ΔC_T_=C_T_ (Target, TLR) - C_T_ (Endo, GAPDH). The comparative ΔΔC_T_ calculation involved finding the difference between ΔC_T_ of treated cells and the mean value of the ΔC_T_ from the untreated cells. Fold increase in the expression of specific mRNA in treated cells compared to untreated cells was calculated as 2^-(ΔΔC^_T_^)^. Data are expressed as RQ (relative quantity) and differences are shown in the figures as the expression ratio of the normalized target gene according to the software results.

### Immunofluorescent staining

HCECs were seeded onto Laboratory-Tek tissue culture chamber slides without FBS for 24 h. The cells were then washed with Hank's Balance Salt Solution (Invitrogen-Gibco) and stimulated with 10 μg/ml hydrocortisone for 48 h. The slides were then fixed in 4% paraformaldehyde for 15 min and washed with 10X Tris Buffered Saline (TBS) three times for 5 min. Fixed cells were incubated in blocking buffer of 5% BSA (Proliant, Ankeny, IA) and 0.1% Triton X-100 in PBS for 30 min at room temperature. Cells were then incubated with the following dilutions of primary antibodies for 1 h at room temperature: primary mouse anti human TLR2 and four monoclonal antibodies (20 μg/ml in 5% BSA-PBS) or with mouse IgG (control). The secondary antibodies that were conjugated to Cy3 were diluted 1:200 in 5% BSA-PBS and incubated for 1 h at room temperature. Coverslips were washed three times in PBS for 5 min, mounted (Vectashield; Vector Laboratories, Burlingame, CA), and viewed with a fluorescence microscope (Zeiss microscope Imager Z1; Zeiss, Oberkochen, Germany). The DNA-intercalating dye, DAPI dihydrochloride, was used to stain nuclei. For the negative control, preimmune mouse serum was substituted for the primary antibody.

### Western blot

Cells challenged with hyphal fragments were lysed in radioimmunoprecipitation (RIPA) buffer (150 mM NaCl, 100 mM Tris-HCl [pH 7.5], 1% deoxycholate, 0.1% sodium dodecyl sulfate [SDS], 1% Triton X-100, 50 mM NaF, 100 mM sodium pyrophosphate, 3.5 mM sodium orthovanadate, proteinase inhibitor cocktails, and 0.1 mM phenylmethylsulfonyl fluoride [PMSF]). Protein concentration was determined using the bicinchoninic acid (BCA) assay (Micro BCA; Pierce Biotechnology, Rockford, IL). Equal amounts of protein were mixed with SDS–PAGE protein loading buffer and boiled for 5 min. Proteins were separated by sodium dodecyl sulfate-PAGE in Tris/glycine/SDS buffer (25 mM Tris, 250 mM glycine, and 0.1% SDS) and electro-blotted onto nitrocellulose transfer membranes. After blocking with 5% nonfat milk for 1 h, membranes were washed three times with TBST for 5 min and incubated overnight with polyclonal antibodies against TLR2 and TLR4 (1:1000 dilution in 5% nonfat milk) in TBST. GAPDH was used as the control. After washing three times in TBST, membranes were incubated with secondary HRP-conjugated anti-mouse IgG for 1 h. The membranes were again washed with TBST three times and one time in TBS, 5 min each. Immune complexes were visualized with an enhanced chemiluminescence reagent (Pierce). Results were quantified by capturing the exposed X-ray film image and using area measurements from image analysis software.

### Enzyme-linked immunosorbent assay

The concentrations of IL-6 and IL-8 in the cell culture supernatants were determined by ELISA. The assay was performed according to manufacturer's instructions. Results from two representative experiments are presented as the means±SEM of triplicate cytokine measurements.

### Statistical analysis

Data are expressed as mean±SEM of triplicates from experiments repeated three times that yielded similar results. Statistical significance of differences was determined with the nonparametric Wilcoxon test and Student's *t* test using SPSS (version 11.5). Differences were considered statistically significant at p<0.05.

## Results

### Expression of toll-like receptors 1–10 mRNAs in human corneal epithelial cells

**Figure 2:**
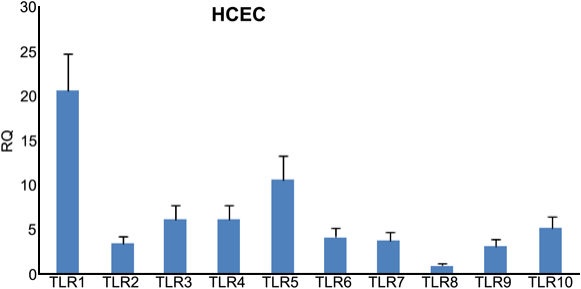
Real time PCR shows the relative expression of TLR1–10 mRNA

Preliminary studies using real time PCR revealed that HCEC expressed varying levels of mRNA for TLR1–10 (Figure 2). TLR1, 3, 4, and 5 were expressed at relatively higher levels in HCECs relative to the other TLRs.

The TLR8 mRNA is regarded as the standard control (RQ=1). Expression of the other TLRs is expressed relative to TLR8. Note that the relative quantity (RQ) is inverted so that higher histogram bars represent higher levels of mRNA expression. Bars represent mean±SEM of three healthy corneas.

### Modulation of toll-like receptor mRNA expression by hyphal fragments from *Fusarium solani*

**Figure 3:**
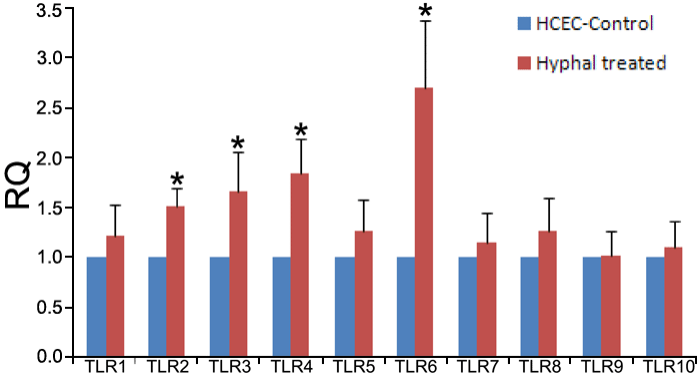
Toll-like receptors 1–10 mRNA expression in hyphal fragment-treated human corneal epithelial cells compared with untreated human corneal epithelial cells

The results of real time PCR indicated that inactive hyphal fragments treatment can increase the mRNA expression of TLR2, 3, 4, and 6 in cultured HCEC (Figure 3).

The expression of each TLR in untreated HCEC is regarded as the standard control (RQ=1), and the TLRs in HCECs treated with hyphal fragments are expressed as the multiple of the untreated HCECs. Bars represent mean±SEM of three independent experiments. The asterisk represents p<0.05 versus control.

### Hydrocortisone can modulate the expression of toll-like receptor mRNA in human corneal epithelial cells

**Figure 4:**
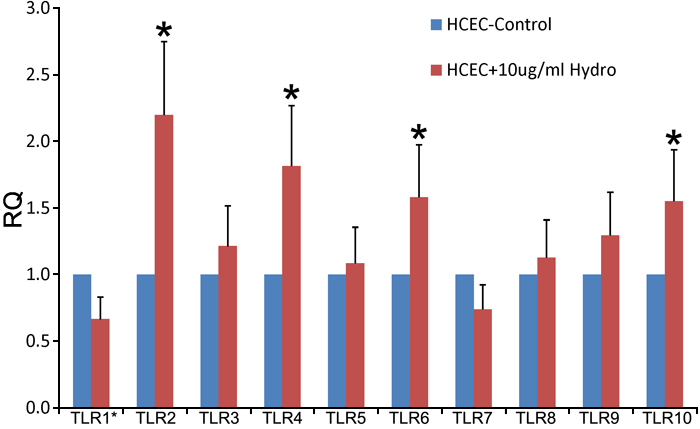
Toll-like receptors 1–10 mRNA expression in hydrocortisone treated human corneal epithelial cells compared with untreated human corneal epithelial cells

**Figure 5:**
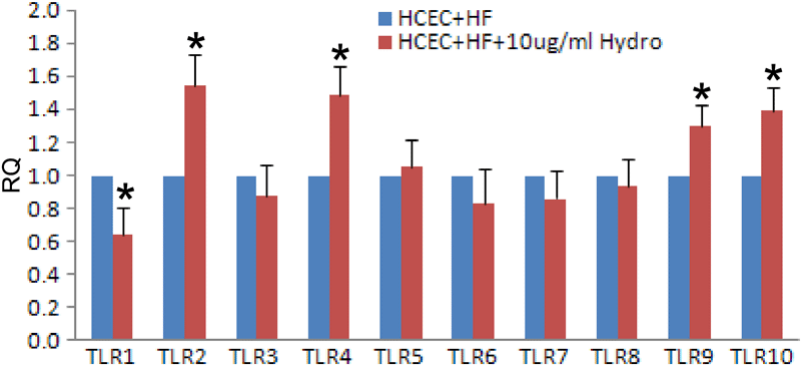
Toll-like receptors 1–10 mRNA expression in hydrocortisone and hyphal fragments-treated human corneal epithelial cells compared with levels in human corneal epithelial cells treated with hyphal fragments alone

The results of real time PCR showed that hydrocortisone treatment upregulated the expression of TLR2, 4, 6, and 10 mRNA in HCECs (Figure 4). The increased transcription of TLR2 and TLR4 was especially pronounced. Hydrocortisone also increased mRNA expression of TLR 2, 4, 9, and 10 in the presence of hyphal fragments (Figure 5).

The expression of each TLR in untreated HCECs is regarded as the standard control (RQ=1), and the TLR expression in hydrocortisone-treated HCECs is expressed as the multiple of the expression of HCEC. Bars represent mean±SEM of three independent experiments. The asterisk represents p<0.05 versus control. Hydro: hydrocortisone.

The expression of each TLR in hyphal fragments-treated HCECs is regarded as the standard control (RQ=1), and the TLRs in both hydrocortisone and hyphal fragments-treated HCEC is expressed as the multiple of the TLR expression in HCECs treated with hyphal fragments alone, Bars represent mean±SEM of three independent experiments. The asterisk represents p<0.05 versus control. Hydro: hydrocortisone.

### Increased expression of toll-like receptor 2 and 4 protein following hyphal fragment treatment

**Figure 6:**
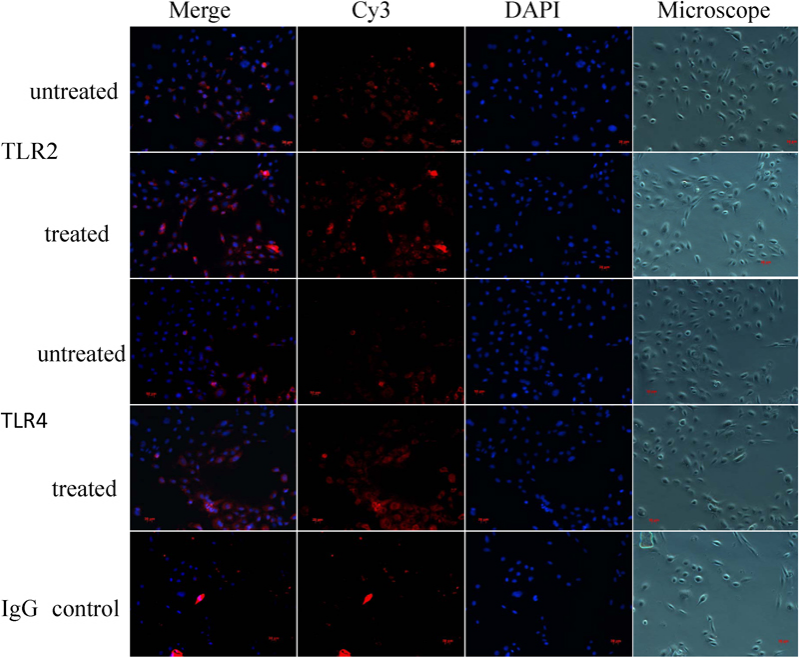
The hyphae fragments-stimulated cells treated with anti-TLR2 and anti-TLR4 antibodies and stained by Cy3 and 2-(4-Amidinophenyl)-6-indolecarbamidine dihydrochloride

The results of immunofluorescence staining revealed moderate TLR2 and 4 reactivity in untreated HCECs. The staining intensity of these antigens was slightly enhanced after treatment with hyphal fragments (Figure 6).

There was no immunoreactivity in the negative control (isotype IgG). Merge means overlap with the DAPI and Cy3.

**Figure 7:**
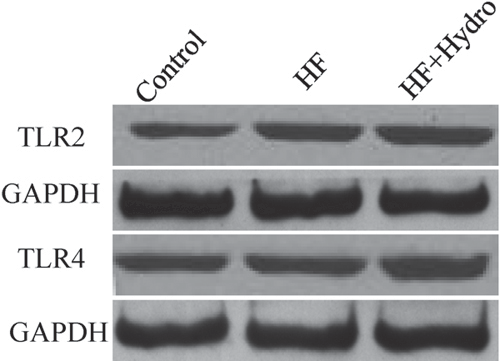
Western blot analyses detect the expression of TLR2 and TLR4 proteins in human corneal epithelial cells following different stimulation

The expressions of TLR2 and TLR4 were also confirmed by western blot analysis. The results of this analysis demonstrated increased expressions of TLR2 and TLR4 following treatment with hyphal fragments (Figure 7). The expressions of TLR2 and TLR4 proteins following treatment with hyphal fragments were further enhanced by cotreatment with hydrocortisone (Figure 7).

Equal amounts of total protein were loaded. The results demonstrated the upregulated expressions of TLR2 and TLR4 under the treatment of hyphal fragments. This upregulation can be further enhanced by hydrocortisone. The results are representative of three independent experiments. Con: control; HF: hyphal fragments; Hydro: hydrocortisone.

### Increased release of IL-6 and IL-8 in human corneal epithelial cells treated with hyphae fragments

**Figure 8:**
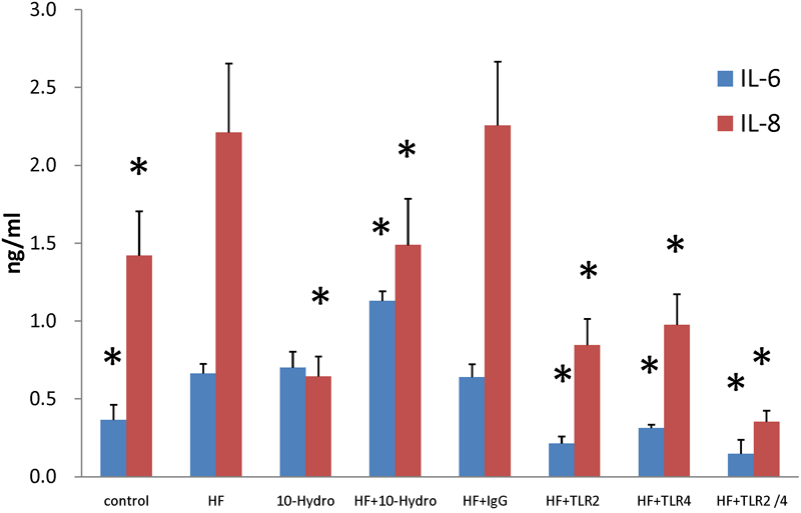
The results of ELISA showed the release of IL-6 and IL-8 from HCEC under different stimulation

Results demonstrate that treatment with hyphal fragments increased the release of IL-6 and IL-8 from HCECs (Figure 8). Hydrocortisone further promoted the release of IL-6 in HCECs treated with hyphal fragments and inhibited the release of IL-8(Figure 8). The results of ELISA assays showed that pretreatment of HCECs with anti-TLR2 or anti-TLR4 inhibited the production of IL-6 and IL-8 following exposure to *Fusarium* hyphae. In contrast, an isotype-matched control, Ab, had no effect on IL-6 or IL-8 production (Figure 8). Maximal inhibition was observed in HCECs treated with antibodies against both TLR2 and TLR4 with a 78% reduction in IL-6 and a 84% reduction in IL-8. In comparison, incubation with TLR2 mAb alone inhibited hyphal-induced IL-6 and IL-8 by 68% and 62%, respectively, while incubation with TLR4 mAb alone inhibited hyphal-induced IL-6 and IL-8 by 53% and 56%, respectively.

Data are the mean±SEM of triplicates from an experiment that was repeated three times with similar results. The asterisk represents p<0.05 versus the corresponding value for cells incubated with hyphal fragments. HF: hyphal fragments; 10-Hydro: hydrocortisone (10 μg/ml); TLR2/4: combined use of TLR2 and TLR4.

## Discussion

A critical component of host defenses against microbes is the ability of the immune system to recognize and respond to foreign invaders. Recent studies have established the central role of TLRs in innate immune recognition of a wide variety of microbial pathogens [30-32]. The current study assessed the contribution of TLR2 and TLR4 to signaling in response to the opportunistic fungus *Fusarium solani*. We demonstrated that hyphal fragments from *Fusarium solani* increased the expression of TLR2, 3, 4, and 6 mRNAs and the release of IL-6 and IL-8. The expressions of TLR2 and TLR4 proteins were also enhanced by exposure to hyphal fragments. The release of IL-6 and IL-8 was also inhibited by pretreatment with anti-TLR2 and anti-TLR4 monoclonal antibodies. Our data suggest that both TLR2 and TLR4 contribute to signaling responses following exposure to *Fusarium solani*.

Corneal epithelial cells are constantly exposed to microbial pathogens and their products on the ocular surface. Although the cornea is highly resistant to infections under normal conditions, sight-threatening microbial infections may occur when the corneal integrity is breached by trauma or contact lens wear. Therefore, the underlying mechanisms that regulate corneal epithelial cell activation are important in the development of infectious keratitis. Previous reports showed that human corneal epithelial cells express functionally active TLR2, 3, and 5 and that these cells respond to *Staphylococcus aureus*, poly(I:C), and *Pseudomonas aeruginosa* and their products, respectively [12-16]. The current study is the first to study the involvement of TLRs in the host response to *Fusarium*. The results of this study demonstrated that exposure of HCECs to inactive hyphal fragment resulted in the upregulation of TLR mRNA expression of TLR2, 3, 4, and 6.

This study focused on TLR2 and TLR4 because TLR3 reportedly only responds to double-stranded (ds) RNA [33,34] and TLR6 mainly recognizes its ligands as a heterodimer with TLR2 [35]. TLR6/TLR2 heterodimers can recognize lipoproteins [35] and peptidoglycan [36]; however, TLR6 has never been reported to play an independent role in the innate immune response. Furthermore, TLR2 and TLR4 have previously been reported to mediate host response to other fungi such as *candidiasis*, *Aspergillus*, and *Cryptococcus neoformans* [17-27].

Results from this study indicated that both TLR2 and TLR4 are likely involved in the host response to *Fusarium* in HCECs. Our results are in agreement with other reports investigating host response to other fungal pathogens. Netea [17] demonstrated that the absence of TLR4-mediated signals resulted in an increased susceptibility to disseminated *candidiasis* in TLR4-defective mice. Experimental models of disseminated *candidiasis* infection in TLR2−/− mice have also shown modulatory effects of TLR2 on host defense [18,19]. Wang [20] proposed that TLR4 but not TLR2 functions as a receptor for *Aspergillus* hyphae. However, other reports have demonstrated that both TLR2 and TLR4 are important for recognition of *Aspergillus fumigatus* [21-24] and *Aspergillus niger* [23]. Other reports have demonstrated that *Cryptococcus neoformans* glucuronoxylomannan binds to TLR2 and TLR4, resulting in translocation of NF-κB caused by the binding of TLR4 but not of TLR2 [25]. More recently MyD88 and TLR2 but not TLR4 were shown to be required for host defense against *Cryptococcus neoformans* infection [26,27].

Glucocorticoids are widely recognized as regulators of adaptive immunity and inflammation. These compounds have been used extensively in clinical settings to suppress a large variety of inflammatory and immune responses. Topical corticosteroids have evolved into the standard treatment for nearly every inflammatory disease of the ocular anterior segment [37,38] and can enhance the invasive abilities of fungi [39]. Despite the importance of glucocorticoids in suppressing immune and inflammatory responses, their role in enhancing host immune and defense responses against invading microbial pathogens is poorly understood. Recent studies by Silverstein [40] and Imasato [41] surprisingly indicate that glucocorticoids could enhance epithelial expression of TLR2 in cultured cells by nontypeable *Hemophilus influenzae* through activation of TLR2 itself [41,42]. This result has recently been confirmed by Homma [43] and suggests that glucocorticoids may enhance the sensitivity of the epithelial surface to TLR ligands in some cases. Therefore, we next investigated whether hydrocortisone can modulate transcription of TLRs in HCECs induced by hyphal fragments. Interestingly, our results showed that hydrocortisone upregulated TLR mRNA especially TLR2 and TLR4. We also demonstrated that TLR2 and TLR4 protein expression was increased in response to hyphal fragments. The results indicate that glucocorticoids enhance the expression of the TLRs on the epithelium and may benefit by inhibiting fungal infections in the epithelium. Thus, it appears that glucocorticoids may not only suppress but also enhance host immune and defense response. In addition, our study may also provide the molecular basis to explain the beneficial role of glucocorticoids in certain cornea infections. However, the exact molecular mechanisms by which glucocorticoids enhance TLR expression in HCECs and contribute to host immune and defense responses remain to be fully elucidated. The synergistic enhancement of TLR expression by glucocorticoids probably contributes to the accelerated immune response of epithelial cells as well as resensitization of epithelial cells to invading pathogens. If so, upregulation of TLRs may be one of the positive immune-regulatory mechanisms involved in glucocorticoid-mediated host defense against many pathogens. In this study, we showed that hydrocortisone can upregulate transcription of TLRs, especially in TLR2 and TLR4, regardless of treatment with hyphal fragments (Figure 4 and Figure 5). The results indicate that the hydrocortisone may enhance the anti-fungal infection immune response of the host by increasing expression of TLRs. Therefore, our study may provide new insights into the role of glucocorticoids in innate immune functions.

Recognition of pathogen-associated molecular patterns (PAMPs) via TLRs can lead to translocation of the nuclear factor NF-κB, resulting in upregulation of proinflammatory cytokines, costimulatory molecules, and chemokines [44,45] such as TNF-α, IL-6, IL-8, IL-18, and monocyte chemotactic protein-1 (MCP). Furthermore, the fact that hyphal fragments induce cytokine production in several leukocyte subsets supports the contention that cytokines play important roles in the host's defense against fungal pathogens [46-48]. In this study, we investigated IL-6 and IL-8 secretion to determine whether the expressions of TLR2 and TLR4 play functional roles. We found that activation of TLRs in response to *Fusarium solani* hyphal fragments resulted in the production of IL-8, the major chemokine that attracts polymorphonuclear leukocytes (PMNs) into the infected cornea, and IL-6, which activates PMNs. A similar study also reported poly (I:C) [16] and lipopolysaccharides (LPS) [49] induced IL-6 and IL-8 expression in human corneal epithelial cells. Therefore, in response to an invading fungal pathogen, HCECs have the capability to recruit PMNs, which are known to be essential in preventing and restricting fungal infection in the cornea.

At the same time, we observed that hydrocortisone can enhance the release of IL-6. This enhancement apparently correlates with the increased levels of the TLR mRNA following hydrocortisone treatment. These results suggested that the action of hydrocortisone on the cornea may be partially mediated via TLRs.

Treatment with hyphal fragments resulted in upregulation of TLR2 and TLR4 and increased the release of IL-6 and IL-8. Therefore, we investigated whether blocking TLR2 and TLR4 with specific monoclonal antibodies (mAbs) can affect the release of IL-6 and IL-8 in HCECs following treatment with hyphal fragments. The results of ELISA showed that pretreatment of HCEC with anti-TLR2 or anti-TLR4 inhibited the production of IL-6 and IL-8 induced by *Fusarium* hyphae while an isotype-matched control Ab was ineffective (Figure 7). Maximal inhibition levels were obtained with both TLR2 and TLR4 mAb. Our findings suggest that signaling in response to hyphal fragments is mediated via both TLR2 and TLR4 in HCECs and that TLR2 may play a greater role than TLR4.

Extrapolation of results in HCECs to the in vivo corneal epithelium may be problematic due to inherent differences between the two systems. For example, transformation by SV40 may significantly affect the expression of TLRs. However, previous studies have shown that HCECs are an excellent cell line in which to study TLR function in the corneal epithelium. The functions of TLRs in response to pathogen-associated patterns such as flagellin, lipopeptides, and poly(I:C) in HCECs are similar to the functions of TLRs in cultured primary human corneal epithelial cells [12,13,15,50].

In summary, our data suggest that TLRs are involved in the cornea response to invasive fungal infections. TLR2 and TLR4 may play crucial roles in signaling in response to *Fusarium* hyphae in HCECs. Glucocorticoids enhance the expression of the TLRs on the epithelium and may promote resistance to fungal infections in the epithelium. These findings may provide crucial information for understanding the immune mechanisms of fungal keratitis and help design new immune therapeutical approaches to fungal keratitis.
